# Onion Bulb Extract Downregulates EGFR/ERK1/2/AKT Signaling Pathway and Synergizes With Steroids to Inhibit Allergic Inflammation

**DOI:** 10.3389/fphar.2020.551683

**Published:** 2020-10-02

**Authors:** Ahmed Z. El-Hashim, Maitham A. Khajah, Khaled Y. Orabi, Sowmya Balakrishnan, Hanan G. Sary, Ala A. Abdelali

**Affiliations:** ^1^Department of Pharmacology & Therapeutics, Faculty of Pharmacy, Kuwait University, Kuwait City, Kuwait; ^2^Department of Pharmaceutical Chemistry, Faculty of Pharmacy, Kuwait University, Kuwait City, Kuwait

**Keywords:** onion (*Allium cepa* Hysam), asthma, signalling, steroids, synergisctic effects

## Abstract

The treatment of allergic diseases, such as asthma, with both conventional and novel therapies presents a challenge both in terms of optimal effect and cost. On the other hand, traditional therapies utilizing natural products such as onion have been in use for centuries with demonstrated efficacy and safety but without much knowledge of their mechanims of action. In this study, we investigated if the anti-inflammatory effects of onion bulb extract (OBE) are mediated *via* the modulation of the EGFR/ERK1/2/AKT signaling pathway, and whether OBE can synergise with steroids to produce greater anti-inflammatory actions. Treatment with OBE inhibited the house dust mite (HDM)-induced increased phosphorylation of EGFR, ERK1/2 and AKT which resulted in the inhibition of HDM-induced increase in airway cellular influx, perivascular and peribronchial inflammation, goblet cell hyper/metaplasia, and also inhibited *ex vivo* eosinophil chemotaxis. Moreover, treatment with a combination of a low dose OBE and low dose dexamethasone resulted in a significant inhibition of the HDM-induced cellular influx, perivascular and peribronchial inflammation, goblet cell hyper/metaplasia, and increased the pERK1/2 levels, whereas neither treatment, when given alone, had any discernible effects. This study therefore shows that inhibition of the EGFR/ERK1/2/AKT-dependent signaling pathway is one of the key mechanisms by which OBE can mediate its anti-inflammatory effects in diseases such as asthma. Importantly, this study also demonstrates that combining OBE with steroids results in significantly enhanced anti-inflammatory effects. This action may have important potential implications for future asthma therapy.

## Introduction

Natural products have been the cornerstone of therapeutic agents for millennia and more recently an important source of therapeutic drugs with unique structural diversity and pharmacological actions (Newman and Cragg, 2016). Many therapeutic agents currently in use in several therapeutic areas such as cardiovascular, oncology, transplantation are natural products or their derivatives such as digoxin, vincristine, and cyclosporine, respectively. However, their use as pharmaceutical agents has waned over the last few decades in the face of advances in combinatorial chemistry and biopharmaceutical technology, the latter supplying the majority of the top ten block buster drugs in the market in 2018 (Brown et al., 2017). Indeed, more than 70% of the world’s population use herb-based medicines for primary healthcare (Newman and Cragg, 2016). A recent study has also reported that approximately 60% of asthma patients in the UK have used herbal remedies for their asthma (Clark et al., 2010). These findings suggest a strong held belief that natural products not only have therapeutic benefit in a wide range conditions, but that they are also safe.

Inflammatory-based diseases, such as asthma, present a global healthcare challenge. Worldwide prevalence of asthma has been estimated to range from 1% to as high as 18% in different populations, affecting up to 300 million people worldwide (Nunes et al., 2017; WHO, 2019) with increasing prevalence particularly among children (GINA, 2019). It is currently the most common chronic respiratory disease in children and costs over a £1 billion per year in some healthcare systems in Europe (Harrison, 2015). There is also good evidence that food allergy and eczema are rising, in parallel to asthma, and have been described as a “second wave” of allergy epidemic particularly in children *(*Prescott and Allen, 2011*)*. While the mechanisms of asthma still remain unclear, it is well recognized that chronic airway inflammatory disease is central to its pathogenesis and is mediated by inflammatory cells such as mast cells and eosinophils and is driven by specific Th2 and Th17 lymphocytes, cytokines, and chemokines (Massoud et al., 2016; Athari, 2019).

There have been several recent studies demonstrating that pathogenic EGF/EGFR-dependent signaling through EGF and other EGFR ligands, such as amphiregulinin, is increased in asthma (Acciani et al., 2016; Ha and Rogers, 2016). EGFR expression has been reported to be weak or absent in healthy individuals but is significantly increased in the airway epithelium of not only asthmatics (Puddicombe et al., 2000; Takeyama et al., 2001a; Takeyama et al., 2001b; Polosa et al., 2002), but also in patients with COPD (de Boer et al., 2006) and cystic fibrosis patients (CF) (Burgel et al., 2007). Furthermore, in a recent clinical study conducted using *ex vivo* lung tissue from patients with COPD, the EGFR inhibitor BIBW 2948 showed some efficacy in inhibiting EGFR phosphorylation and a tendency toward reducing mucous cell metaplasia. More importantly, a positive correlation between EGFR immunoreactivity and MUC5AC mucin staining was noted when bronchial biopsies from healthy volunteers and subjects with mild-to-moderate asthma were compared, suggesting a causal relationship (Puddicombe et al., 2000). Also, areas of epithelial damage in asthmatic patients exhibited a strong EGFR immunoreactivity suggesting that EGFR activation plays an important role in the epithelial damage/repair process in asthma (Puddicombe et al., 2000). Of interest also is that a positive correlation between mucin and EGFR staining has been shown in the small airway of CF patients (Burgel et al., 2007). Thus, increased EGFR expression is a consistent finding not only in asthma but across several disease states. Moreover, preclinical animal models have also demonstrated a strong role for EGFR in asthma. We and others have shown, using an allergic model of inflammation, that EGFR inhibitors, such as AG1478 or gefitnib, significantly reduce eosinophil recruitment, airway inflammation, airway hyperresponsiveness (AHR), and goblet cell hyper/metaplasia, thus, underscoring the importance of this signaling pathway in asthma pathogenesis (Tamaoka et al., 2008; Le Cras et al., 2011; Song H. N. et al., 2016; El-Hashim et al., 2017). Furthermore, we have also reported that ERK1/2 and AKT are downstream signaling molecules of EGFR activation (El-Hashim et al., 2017). Therefore, both clinical and preclinical studies clearly establish an important role for EGFR-dependent signaling in inflammatory-based diseases.

While the combination of inhaled corticosteroids (ICS) and long-acting beta-agonists (LABA) is a main treatment advocated by most asthma guidelines (O’Byrne et al., 2001; GINA, 2019), a significant number of patients are poorly compliant with inhaled treatments and remain under-controlled (Sherman et al., 2001). Furthermore, severe asthmatics require treatment with moderate to high doses of steroids (Carr and Kraft, 2017). This is associated with a significant side effect profile (Pinto et al., 2013). Therefore, it would be more advantageous if the asthma therapeutic goals can be achieved at lower doses of steroids since the side effects would be minimal. Synergism is a phenomenon whereby combination of drugs produces a greater effect than when each drug is given alone (Jia et al., 2009). This phenomenon is useful when low doses of efficacious drugs are combined as they produce a superior effect but with fewer side effects. Indeed, this has been demonstrated with ICS and several drug classes such as LABA and anti-leukotrienes (Beasley et al., 2019). However, no study has tested if synergism occurs between steroids and natural products within the context of inflammatory based diseases such as asthma.

Novel monoclonal antibody-based therapy, in inflammatory disease management, has made an impact on disease control. For example, the anti-IgE antibody, omalizumab, has been used as a steroid sparing drug in patients with severe asthma, but unfortunately its use is limited (Plosker and Keam, 2008) due to a high frequency of anaphylactic reaction and serum sickness (Harrison et al., 2015) and lack of cost-effectiveness. Similarly, the use of the newly introduced anti-IL5 antibodies such as mepolizumab and benralizumab is limited to severe asthmatics with a high eosinophilic component. However concerns have been raised regarding their cost-effectiveness (Agache et al., 2020). Despite the recent market increase in therapeutic agents that selectively target specific molecules, it is unlikely that the blockade of individual mediator signaling pathways would result in optimal therapeutic outcomes in asthma, and monoclonal based therapy would certainly be beyond the financial reach of most asthma patients in the developing world due to their high cost.

*Allium cepa* L. (Family Amaryllidaceae) is one of the most commonly consumed vegetables and has also been used for medicinal purposes for numerous ailments such as ulcer wounds, scars, dysentery, inflammation, hypertension and also in respiratory conditions such as cough, asthma, and bronchitis (Lanzotti, 2006; Upadhyay, 2016; Zeng et al., 2017). Onion bulb extract (OBE) has also been shown to effectively reduce airway inflammation, IL4, and IgE levels and induces oxidation in animal models of asthma (Oliveira et al., 2015; Marefati et al., 2018). Thiosulfinates (TS) and cepaenes (CS), isolated from onions and/or synthesized, were also shown to have dose-dependent inhibitory effects on both cyclooxygenase and 5-lipoxygenase activity (Dorsch et al., 1990; Wagner et al., 1990) and inhibit *in vitro* chemotaxis of human granulocytes induced by formyl-methionine-leucine-phenylalanine (WKYMvm) (Dorsch et al., 1990). In this regard, it is of interest to note that many of the previous studies that assessed the effects of onion extract have used ovalbumin as the allergenic material to induce airway inflammation. However, the use of ovalbumin has recently been questioned on the basis that it is not clincally relevant, and therefore studies using allergens such as house dust mite or *Alternaria alternata*, that simulate clincal asthma more closely (Gorska, 2018), are necessary in order to better assess the effects of onion extract in animal models.

The EGFR/ERK1/2/AKT signaling pathway has been recently shown to be an important signaling pathway, in both clincial and preclinical studies. However, whether the EGFR/ERK1/2/AKT signaling pathway is a target for OBE in a clincially relevant animal model of allergic asthma, is not known. Also whether OBE synergizes with steroids has not been studied previously. In this study, using a clincially relevant allergen (HDM), we investigated 1) whether the EGFR/ERK1/2/AKT signaling pathway is modulated by OBE, and 2) if OBE synergizes with dexamethasone to result in a greater anti-inflammatory action.

## Methods

### Acquisition of the Plant Material

Fresh red onion bulbs were bought from the local market. The plant was identified as *Allium cepa*, and a voucher specimen, number KOE-010, was deposited at the herbarium of Kuwait University (KTUH), College of Science, Kuwait.

### Extraction of Onion Bulbs

About 20 kg of fresh red onion was peeled, coarsely cut, and percolated three times, each using 10 L of dichloromethane. The dichloromethane layers of the percolates were separated from the aqueous layer, dried over anhydrous sodium sulphate, and then evaporated in *vacuo* till dryness to obtain brownish syrupy residue. This extraction process was repeated as needed. The treatment stock solution was prepared using the residue and PBS as a vehicle.

### GC–MS Analysis

Dichloromethane extract of onion bulbs (1 μl sample size) was analyzed on a Thermo high resolution gas chromatography**–**mass spectrometer Double Focusing Sector system (GC**–**MS DFS) fitted with a DB-5MS capillary column with 0.25 μm film thickness, 30 m length and 0.25 mm inner diameter, using helium as a carrier gas with a flow rate of 0.8 ml/min. The operating conditions were as follows: splitless injector with port temperature 250°C, detector temperature 280°C, and program temperature from 50 to 250°C at the rate of 6°C/min with 10 min hold time, and from 250 to 300°C at the rate of 10°C/min with 6 min hold time. The MS conditions were as follows: electron impact ionization mode, ionization energy 70 eV, ion source temperature 175°C, scan range *m/z* 40–900 Da. The qualitative identification of the compounds was based on computer matching with NIST MS Search 2.0 library and by comparison with data in the literature (Mondy et al., 2001; Gitin et al., 2014; Abdel-Lateef et al., 2018; Fredotovic et al., 2020).

### Animals

Male BALB/c mice (6–8 weeks old, average weight 25 g) were used in this study. All studies involving animals were in accordance with the ARRIVE guidelines for reporting experiments involving animals. All experimental protocols were approved by the Animal Welfare and Use of Laboratory Animals Committee in the Health Sciences Center, Kuwait University and were carried out in accordance with the EU Directive 2010/63/EU for animal experiments and the National Institutes of Health guide for the care and use of Laboratory animals (NIH Publications No. 8023, revised 1978). Animals were maintained under temperature-controlled conditions with an artificial 12 h light/dark cycle and were allowed standard chow and water *ad libitum*.

### Immunization and Intranasal Challenge and Drug Treatment Protocols

#### Protocol for Prophylactic Treatment Experiments

**Figure 1:**
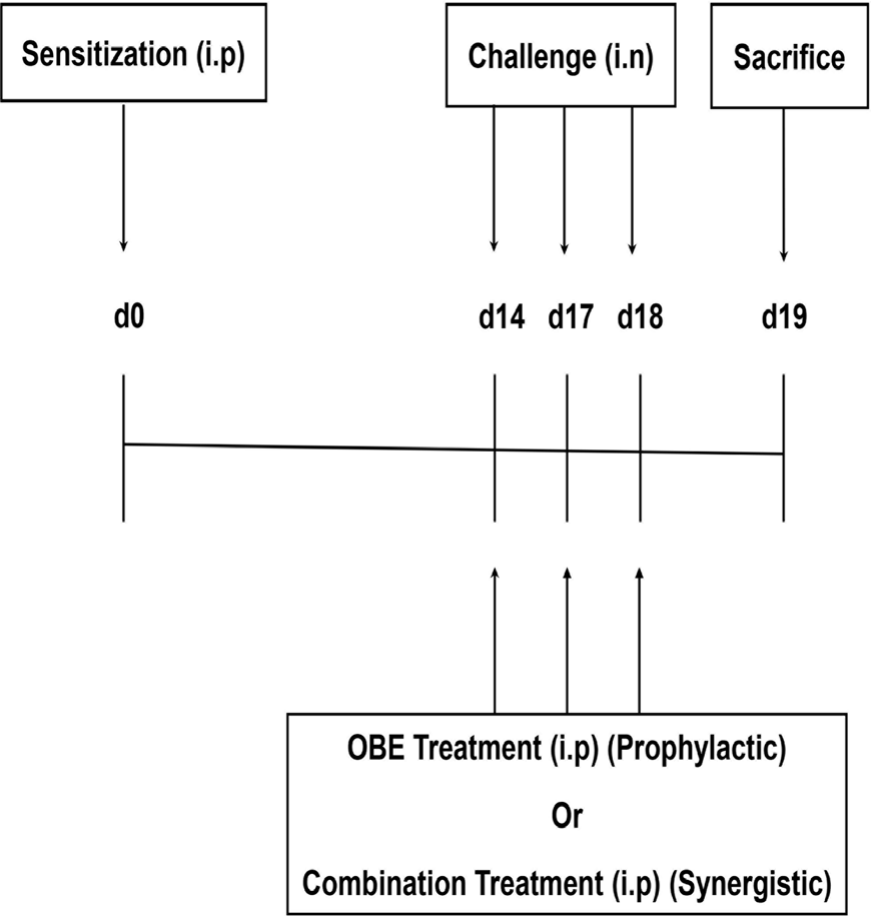
Schemactic representation of the immunization and the treatment protocol used for boh the prophylactic and synergistic studies.

Seven treatment groups (A–G, 9–15 animals per group) were established to determine whether OBE if given prophylactically inhibits the HDM-induced asthma phenotype. All mice were immunized once by intraperitoneal (i.p.) injection of 40 µg HDM in 0.2 ml of alu-Gel-S (Alu-Gel-S; SERVA Electrophoresis GmbH) on day 0. Mice were subsequently challenged for 3 days, days 14, 17, and 18 with HDM or PBS in the case of the control group. Mice in groups A (n = 11) and B (n = 13) were pretreated intraperitoneally with 0.2 ml of the drug vehicle, 1 h before each intranasal challenge with PBS and HDM, respectively. In the same manner, groups C (n = 10), D (n = 9), E (n = 10), and F (n = 15) were pretreated with the same volume of OBE at 10, 30, 60, and 100 mg/kg, respectively, and group G (n = 11) with dexamethasone (3 mg/kg), 1 h before each intranasal challenge with HDM (**Figure 1**).

24 h after the last intranasal challenge, animals were sacrificed with an overdose of halothane; bronchoalveolar lavage (BAL) was performed to obtain BAL fluid, and then the lungs were excised for preparation for histology, Western Blot (WB), and immunofluorescence (IF) studies. In another separate group of animals, airway responsiveness was measured. For the histology/WB/IF studies, the OBE dose of 60 mg/kg was selected to represent the prophylactic approach as it gave an optimum effect.

#### Protocol for Synergistic Treatment Experiments

Six treatment groups (A–F, 9-11 animals per group) were established to determine whether combining OBE and dexamethasone would results in synergistic anti-inflammatory actions. Mice in groups A (n = 9) and B (n = 11) were pretreated intraperitoneally with 0.2 ml of the drug vehicle 1 h before each intranasal challenge with PBS and HDM, respectively. In the same manner, groups C (n = 11), D (n = 7), E (n = 11), and F (n = 9) were pretreated with the same volume of OBE at 30 mg/kg (OBE 30), OBE at 30 mg/kg in combination with dexamethasone (DEX) at 0.5 mg/kg (OBE 30 + DEX 0.5), 0.5 mg/kg (DEX 0.5 mg) and 3 mg/kg (DEX 3 mg), respectively, 1 h before each intranasal challenge with HDM. Treatment with OBE and/or dexamethasone was also repeated on days 14, 17, and 18 (**Figure 1**).

### Bronchoalveolar Lavage Fluid Cell Counts and Lung Histology

BAL fluid was collected by cannulating the trachea and washing the lungs with saline solution (0.3 ml × 4 each) after sacrificing the animals with an over dose of halothane. The BAL cells were then counted using a particle-size counter (Z1 Single Threshold; Beckman Coulter), and cytosmears were prepared for differential count. The cells were stained with Diff-Quik, and a differential count of 200 cells was performed using standard morphologic criteria. Results are expressed as total cell count/ml and as total macrophages, lymphocytes, neutrophils, and eosinophils/ml in BAL fluid. For histological assessment, segments of the lung tissue were removed and fixed in 10% buffered formalin, embedded in paraffin wax and sectioned into 5-µm-thick slices. The sections were processed and stained separately with H&E stain and periodic acid–Schiff (PAS) according to standard methods as previously described (El-Hashim et al., 2017). Sections were examined under light microscope and the severity of pathologic changes scored independently by two experienced histologists unfamiliar with the slides. Score coding was as follows: (1 = normal, 2 = mild, 3 = moderate, 4 = severe and 5 = highly severe).

### Measurement of Airway Responsiveness

Airway responsiveness was measured 24 h after last HDM or PBS challenge, using a separate set of animals using the Buxco FinePointe series RC site (DSI, Wilmington, NC) as described previously (Queto et al., 2010; Ezeamuzie et al., 2014; Correa et al., 2017). Briefly, mice were anesthetized with an intraperitoneal injection of ketamine/xylazine (1:0.1 mg/kg) cocktail and tracheotomized with a steel 18-gauge cannula. Mice were then mechanically ventilated at a rate of 150 breaths/min and a tidal volume of 0.15 ml using a computerized small animal ventilator (FinePointe site). Following a 5 min period of stabilization and administration of PBS, airway resistance was measured by exposing mice to aerosolized methacholine (6.25–50.0 mg/ml, 5 µl per delivery) delivered by an aerogen nebulizer and reported as total lung resistance (*R*_L_) (cm H_2_O per ml/s).

### Immunofluorescence

Lung tissues were processed as described above. Immunofluorescence was performed as previously described (Khajah et al., 2016). In short, lung sections were incubated in a blocking solution (5% bovine serum albumin (BSA) + 0.3% Triton X-100 in PBS) for 1 h and were subsequently incubated overnight at 4°C with primary antibodies [p-EGFR (Tyr1068) (Rabbit; Cat. No. 3777S), pAKT (Ser 473) (Rabbit; Cat. No. 9271L) and pERK1/2 (Thr202/Tyr204) (Rabbit; Cat. No. 9101L) (1:25–1:800 dilution) or only 1% BSA (for negative control); Cell Signaling, USA], diluted in 1% blocking solution. 24 h later, sections were washed and incubated with secondary antibody conjugated to Alexa Fluor 555 (Goat anti rabbit SFX kit; Life Technologies, USA, 1:400 dilution) for 2 h at room temperature in the dark. Following several washes in PBS, sections were stained with 4′, 6 diamidino-2-phenylindole and mounted. Images were then captured on a ZEISS LSM 700 confocal microscope and fluorescence intensity estimated in defined fields using Image J software package. The laser setting and photo processing were equal among the different treatment groups for each protein. 40× magnification for the tested molecules was equally modified in terms of sharpness and contrast to show localization of the phospho-proteins in the lung tissue.

### Western Blotting

Appropriate lobes from the dissected lungs of the mice were snap-frozen in liquid nitrogen and stored at −80°C. Following that, the tissue samples were defrosted in ice then transferred to lysis buffer (pH 7.6) containing 50 mM Tris-base, 5 mM EGTA, 150 mM NaCl, 1% Triton 100, 2 mM Na_3_VO_4_, 50 mM NAF, 1 mM PMSF, 20 µM phenyl arsine, 10 mM sodium molybdate, 10 µg ml^–1^ leupeptin and 8 µg ml^−1^ aprotinin. Using a homogenizer, the tissues were homogenized for 10 s, three times. Samples were allowed to lyse completely by incubation on ice for 30 min. The lysates were then centrifuged at 13,000 rpm for 10 min at 4°C and the supernatants collected, and protein concentrations were estimated by Bio-Rad Bradford Protein Assay (Bio-Rad, Hercules, CA, USA). Aliquots containing equal amounts of protein were subjected to SDS-PAGE and transferred electrophoretically onto nitrocellulose membrane (Schleicher & Schuell, Dassel, Germany). The membranes were blocked with 5% BSA and then incubated with ERK1/2 (137F5) (Rabbit; Cat. No. 4695S), pERK1/2 (Thr202/Tyr204) (Rabbit; Cat. No. 9101L) and β-actin antibody (Cell Signaling Technology, Boston, MA, USA; 1/1,000 dilution) (used as loading control, 1:1,000 in 5% BSA) at 4°C overnight. Membranes were incubated with appropriate secondary antibodies conjugated to horseradish peroxidase (Amersham, Buckinghamshire, UK) to detect phosphorylated form of ERK1/2 (42/44 kDa), or total form of actin (45 kDa). The immunoreactive bands were detected with Super Signal Chemiluminescent Substrate (Immuno Cruz Western blotting luminal reagent SC-20428, Santa Cruz Biotechnology) utilizing a Kodak autoradiography film (Care stream Biomax Xarfil 1660760). Images were then analyzed and quantified and the data were normalized to *β*-actin levels. The experiment was run twice with lung samples from three different mice in each treatment group (pooled) in each run.

### Measurement of Lung Cytokines

Lung tissues from mice were collected and stored at −80°C. The amount of total protein was determined by Bradford analysis using the Bio-Rad Protein Assay reagent. The relative changes of different cytokines and chemokines were detected using Proteome Profiler^™^ Mouse Cytokine Array Kit (Catalog # ARY006, R&D Systems, Inc., Minneapolis, USA). The procedure was done in line with the manufacturer’s protocols and as recently described (Khajah et al., 2019).

### Isolation of Human Blood Eosinophils

For this experiment, fresh blood was obtained from healthy individuals with no history of allergic disease and had not taken any medication in the last 72 h after receiving their informed consent. The methods and protocol for these experiments were performed in accordance with and approved by the “Ethical Committee of the Faculty of Medicine, Kuwait University”. Granulocytes were isolated from heparinized (10 IU/ml) blood by erythrocyte sedimentation, followed by percoll gradient centrifugation as reported recently (Ezeamuzie et al., 2014). Eosinophils were separated using negative selection with the immunomagnetic method as previously described (Hansel et al., 1991). The eosinophil purity was checked by differential count of a Wright–Giemsa stained cytosmear and was routinely >98%. Viability was determined by Trypan blue exclusion and exceeded 98%.

### Boyden Chamber Assay for Eosinophil Chemotaxis

Peripheral blood derived eosinophils were used for chemotaxis assay utilizing a Boyden chamber as previously described (Gomez-Cambronero et al., 2003). Purified naïve eosinophils (2 × 10^5^) were then placed in the upper wells, and 500 µl of BAL fluid derived from mice challenged with PBS (vehicle) or with HDM, pretreated *ex-vivo* with either vehicle or OBE (100 and 1,000 ng/ml), were placed in the lower wells (37 °C/5%CO_2_) and eosinophils allowed to migrate for 1 h. The transmigrated eosinophils were determined by counting under the microscope by using a hemocytometer.

### Statistical Analyses

All numerical values were expressed as means ± SEM. Total cell counts represent the number of cells/ml of BAL fluid. Differential cell counts represent the absolute number of each cell type/ml of BAL fluid. Absolute R_L_ values were computed and were used as an index of airway responsiveness. For the histopathological assessment, a semi-quantitative 5-level lung pathology score was used to grade the degree of inflammation in each microscopic field at 20×. All data were initially assessed for normality. One-way analysis of variance (ANOVA) test followed by Bonferroni *post hoc* was used to compare differences between individual groups for both total and differential cell count as well as histopathological data and the immunofluorescence data for both the prophylactic and the synergistic studies. A two-way repeated measure analysis of variance followed by a Bonferroni *post hoc* test was used for the airway responsiveness data. The mean difference was considered as significant at a probability level of less than 0.05. All analyses were performed using GraphPad Prism.

## Results

### Extraction of Onion Bulbs

**Figure 2:**
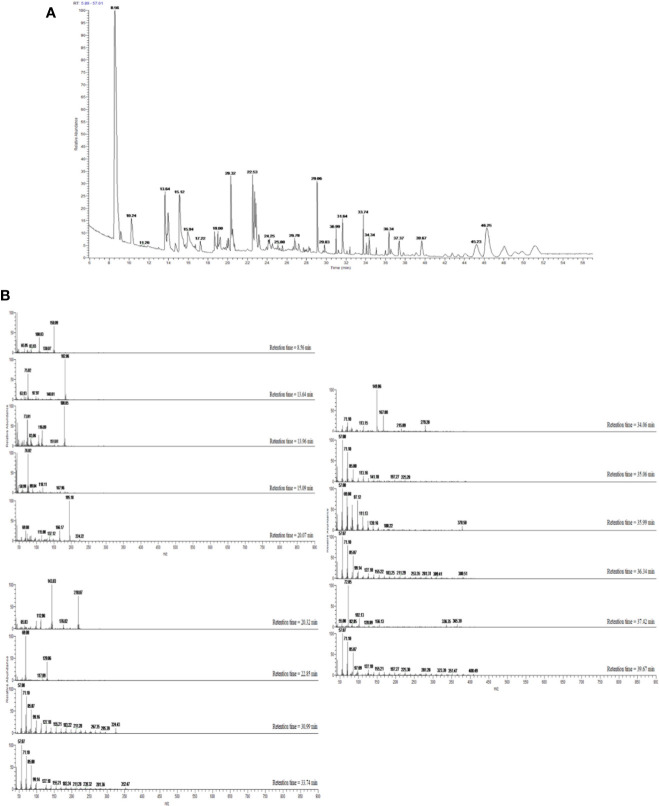
**(A)** GC–MS total ion chromatogram (TIC) of OBE. Peaks were identified through comparison with mass spectral data in the NIST MS Search 2.0 library stored in the GC–MS system and are represented in **Table 1**. **(B)** Mass spectra of compounds eluted with retention times 8.56–46.25 min and presented in **Table 1**.

The extraction process was repeated four times to afford 7.5 g (0.038% yield) of brownish syrupy residue (**Figure 2**).

### GC–MS Analysis of OBE

Dichloromethane extract of *Allium cepa* bulb (OBE) was analyzed to identify different compounds and confirm the identity of the plant. The identified compounds and their mass spectral data are listed in **Table 1**. The major compound identified in the extract was shown to be the sulfur-containing compounds dipropyl disulfide, dipropyl trisulfide, and propylpropane thiosulfonate. Other sulfur compounds were also identified but at lesser quantities. **Figure 2A** shows the TIC of OBE where different 28 peaks are shown. Furthermore, **Figure 2B** shows the fragmentation patterns of some of these compounds. The identity of these compounds was confirmed *via* a direct comparison and matching with the stored spectra in the GC–MS system.

**Table 1:** Compounds identified in *Allium cepa* bulb extract.

Identified Compounds*^a^*	Retention timet_r_ (min)	Area (%)
Dipropyl disulfide	8.56	45.93
Dipropyl trisulfide	13.64	3.16
3,5-Diethyl-1,2,4-trithiolane	13.96	1.88
Propylpropane thiosulfonate	15.09	5.07
1,5-Dithiaspiro[5.6]dodecan-7-ol	20.32	3.46
Methyl-2,6-anhydro-3,4,7-tridesoxy-1-erythro-hept-2-enulonate	22.85	1.25
Tricosane	30.99	0.63
Pentacosane	33.74	1.37
1,2-Benzenedicarboxylic acid	34.06	0.34
Hexacosane	35.06	0.3
1-Heptacosanol	35.99	0.18
Nonacosane	36.34	1.2
Nonacosane	39.67	1.88
Tetratriacontane	45.23	4.09

a Identified by matching with mass spectral data in the NIST MS Search 2.0 library stored in the GC-MS system, and comparison with data reported in the literature.

### Effect of OBE on HDM-Induced Inflammatory Cell Influx

**Figure 3:**
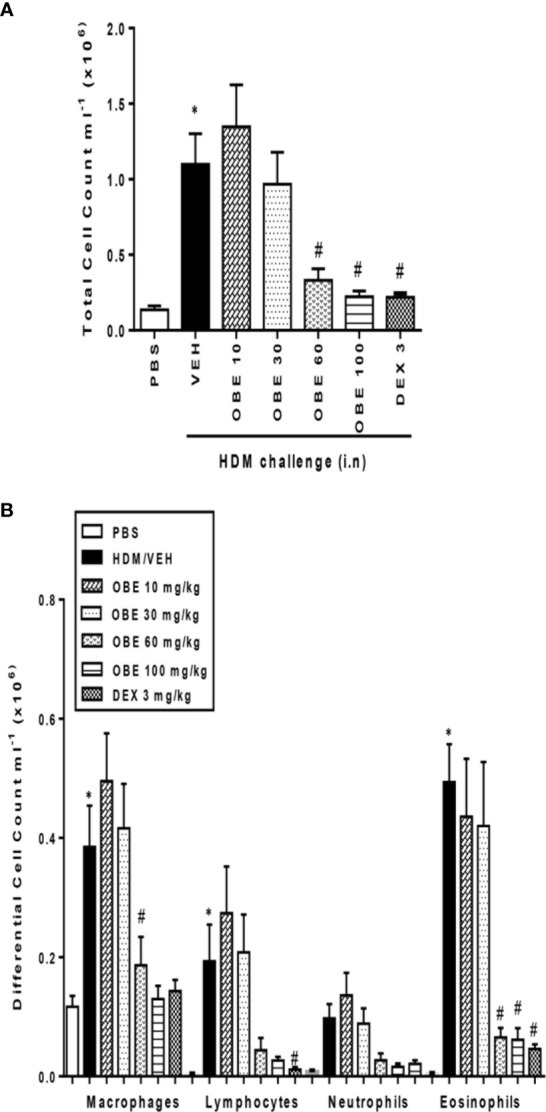
Effect of OBE, 10, 30, 60, and 100 mg/kg (i.p.) on HDM-induced increase in **(A)** total cell and **(B)** differential cell count. OBE treatment resulted in a dose-dependent inhibition of total and eosinophil numbers. Data are expressed as mean ± SEM (*n* = 9–15) *P < 0.05 *vs* PBS group, ^#^P < 0.05 *vs* HDM group.

In these experiments, we evaluated the effect of OBE on HDM-induced total and differential cell influx. Our findings show that HDM-sensitized and challenged animals (HDM group) developed a significant increase in total cell count (1.3 ± 0.3 *vs* 11.0 ± 2.0 × 10^5^ cells/ml) as well as in lymphocytes (0.03 ± 0.01 *vs* 1.9 ± 0.6 × 10^5^ cells/ml) and eosinophils (0.04 ± 0.02 *vs* 4.9 ± 0.6 × 10^5^ cells/ml), (P < 0.05; **Figures 3A, B**, n = 9–15), 24 h after the last HDM challenge compared to the control group. Prophylactic treatment with OBE (10, 30, 60, and 100 mg/kg) dose-dependently inhibited the HDM-induced increase in the total cells and was significant at the doses of 60 and 100 mg/kg (3.3 ± 0.7 and 2.2 ± 0.4 *vs* 11.0 ± 2.0 × 10^5^, respectively, P < 0.05; **Figure 3A**, n = 9–15) and were comparable to the dexamethasone (DEX) group (used as a positive control). Moreover, OBE treatment also inhibited the HDM-induced increase in lymphocytes (0.4 ± 0.2 *vs* 1.9 ± 0.6 × 10^5^ cells/ml, P < 0.05; **Figure 3B**, n = 9–15) and eosinophils (0.6 ± 0.2 *vs* 4.9 ± 0.6 × 10^5^ cells/ml, P < 0.05; **Figure 3B**, n = 9–15).

### Effect of OBE on HDM-Induced Histopathological Changes

**Figure 4:**
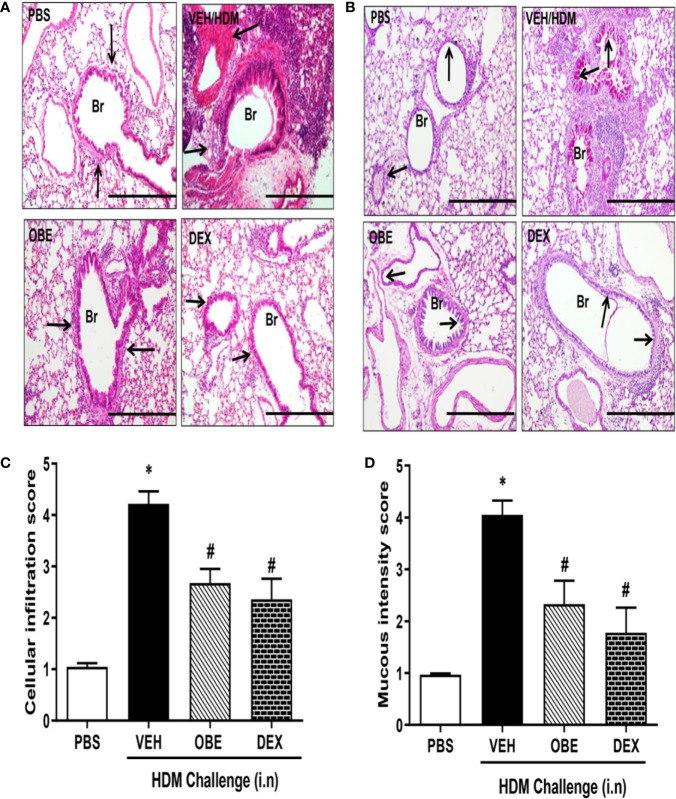
Effect of OBE (60 mg/kg; i.p.) on HDM-induced histopathological changes: **(A)** representative low-magnification light photomicrographs displaying **(A)** H&E and **(B)** PAS staining of whole lung samples from control PBS-challenged mice (PBS), HDM-challenged mice (HDM), HDM-challenged mice pretreated with OBE (60 mg/kg; i.p.) (OBE) and HDM-challenged mice pretreated with dexamethasone (3 mg/kg; i.p.) (DEX), scale bar = 200µm. Graphs shows **(C)** cellular infiltration and **(D)** mucous intensity score for H&E and PAS staining, respectively. OBE treatment resulted in a significant decrease in both peribronchial and perivascular inflammatory cell infiltrations and bronchial mucus production and goblet cell hyper/metaplasia compared with HDM-challenged vehicle treated mice. Data are expressed as mean ± SEM (*n* = 3–6). *P < 0.05 *vs* PBS group, ^#^P < 0.05 *vs* HDM group.

H&E and PAS stained lung sections from control mice (PBS group) showed normal histology (**Figures 4A, B**). In contrast, lung sections from mice challenged with HDM showed consistently marked and severe perivascular and peribronchial inflammatory cell infiltration (cellular infiltration score, HDM vs PBS, 4.2 ± 0.2 *vs* 1.0 ± 0.07) and increase in bronchial mucus production and goblet cell hyper/metaplasia (mucous intensity score, HDM *vs* PBS, 4.1 ± 0.3 *vs* 1.0 ± 0.02) demonstrating a marked degree in airway remodeling (P < 0.05; **Figures 4A–D**, n = 3–6). However, lung sections from OBE-treated mice (60 mg/kg) showed a significantly lower score of the histopathological parameters that were assessed (cellular infiltration score; 2.7 ± 0.3 *vs* 4.2 ± 0.2, and mucous intensity score; 2.3 ± 0.5 *vs* 4.1 ± 0.3), achieving almost normal histological appearance that was very similar to the dexamethasone treatment group (P < 0.05; **Figures 4A–D**, n = 3–6).

### Effect of OBE on HDM-Induced Phosphorylation of EGFR, ERK1/2, and AKT as Determined by Immunofluorescence

**Figures 5:**
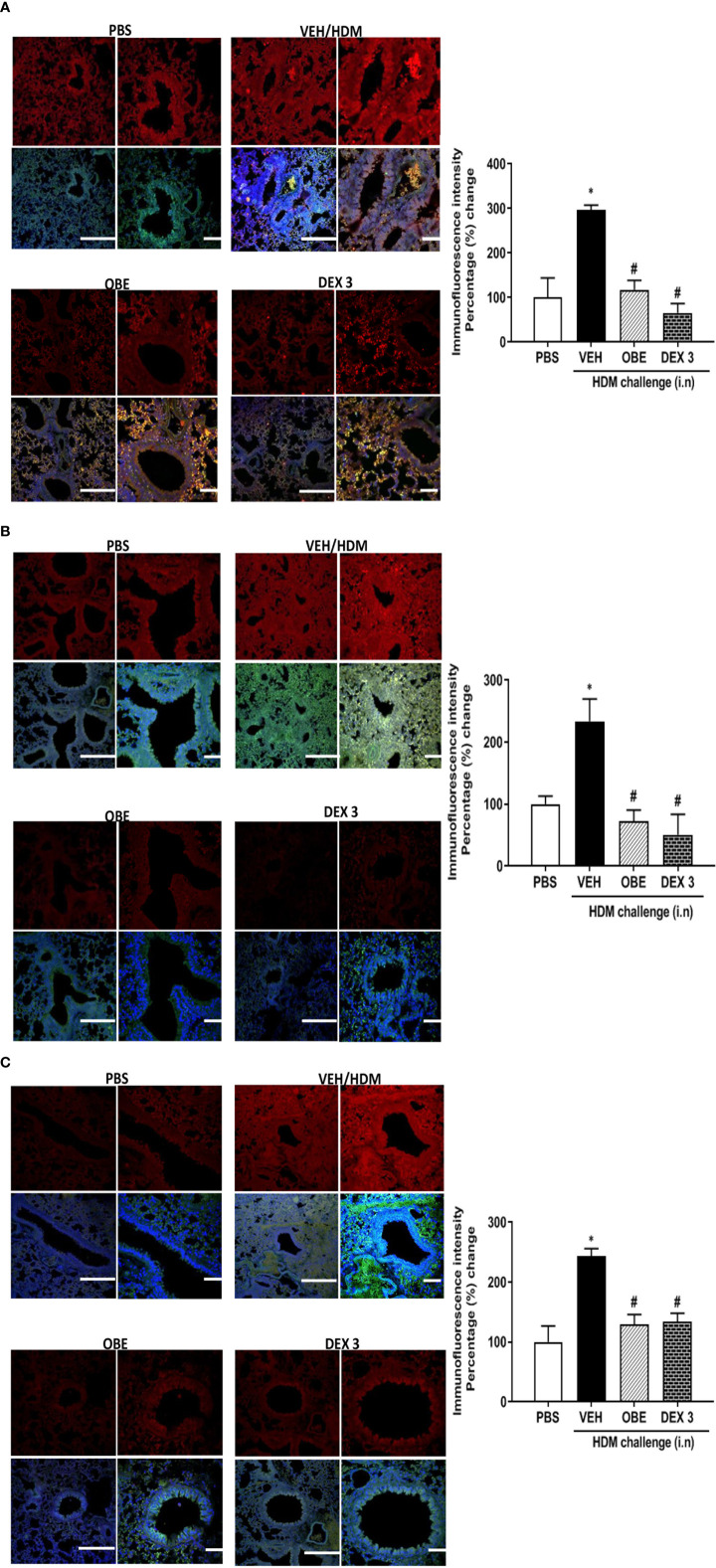
Immunofluorescent (Alexa Fluor) detection of pEGFR **(A)** pERK1/2 **(B)** and pAKT **(C)** shown in the upper panels overlaid with DAPI stain on the lower panel to show lung tissue architecture. Lung sections were taken from different treatment groups: PBS-challenged mice (PBS), HDM-challenged mice pretreated with vehicle (HDM), HDM-challenged mice pretreated with OBE (60 mg/kg; i.p.) (OBE), and HDM-challenged mice pretreated with dexamethasone (DEX) and immunostained for pEGFR, pERK, and pAKT. PBS-treated mice showed minimal pEGFR, pERK1/2, and pAKT expression. HDM challenge resulted in a significant increase in pEGFR, pERK, and pAKT expression, and this was inhibited following treatment with OBE (60 mg/kg; i.p.) and was comparable to the dexamethasone-treated animals, scale bar = 50 µm. Graphs show quantitative assessment of fluorescence intensity of pEGFR, pERK1/2, and pAKT (arbitrary units). Data are expressed as mean ± SEM (*n* = 3–5). *P < 0.05 *vs* PBS group, ^#^P < 0.05 *vs* HDM group.

Our findings show that HDM challenge induced a significant increase of about 3.0, 2.3 and 2.4-fold in the phosphorylation of EGFR, ERK1/2, and AKT, respectively, compared to PBS control as detected by immunofluorescence (P < 0.05; **Figures 5A–C**, n = 3–5). Negative control shows no non-specific staining (data not shown). Treatment with OBE (60 mg/kg) significantly inhibited the HDM-induced phosphorylation of all proteins (P < 0.05) and was comparable to the inhibition obtained in the dexamethasone treatment group (P < 0.05, **Figures 5A–C**, n = 3–5).

### Effect of OBE on HDM-Induced Phosphorylation of ERK1/2 as Determined by Western Blotting

**Figure 6:**
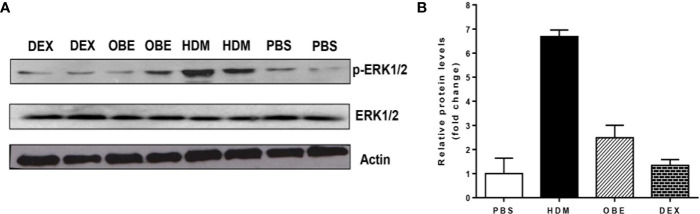
**(A)** Western blot analysis of pERK1/2 and total ERK1/2 protein levels from lungs of PBS-challenged mice pretreated with vehicle (PBS), HDM-challenged mice pretreated with vehicle (HDM), HDM-challenged mice pretreated with OBE (60 mg/kg; i.p.) (OBE) and HDM-challenged mice pretreated with dexamethasone (DEX). The blots are of two pooled lung sample (n = 3, for each). **(B)** Graph b shows relative densitometric quantification levels of pERK1/2 (relative to total ERK1/2, both normalized to *β*-actin).

In this experiment, we assessed the levels of p-ERK1/2 and total ERK1/2 by Western blotting in order to confirm and validate the immunofluorescence data. Western blotting analysis of lung homogenate (**Figure 6A**) confirmed the modulated levels of p-ERK1/2 as seen in the immunofluorescence. HDM challenge resulted in a marked increase in p-ERK1/2 levels compared to PBS-challenged mice (**Figures 6A, B**). Treatment with OBE resulted in a clear inhibition of the p-ERK1/2 and was similar to the dexamethasone-treated group (**Figures 6A, B**, n = 3 for each blot). The effect on HDM and OBE on total ERK1/2 was relatively unchanged.

### Effect of OBE on Airway Levels of Various Cytokines Using a Proteome Profiling-Based Technique

**Figure 7:**
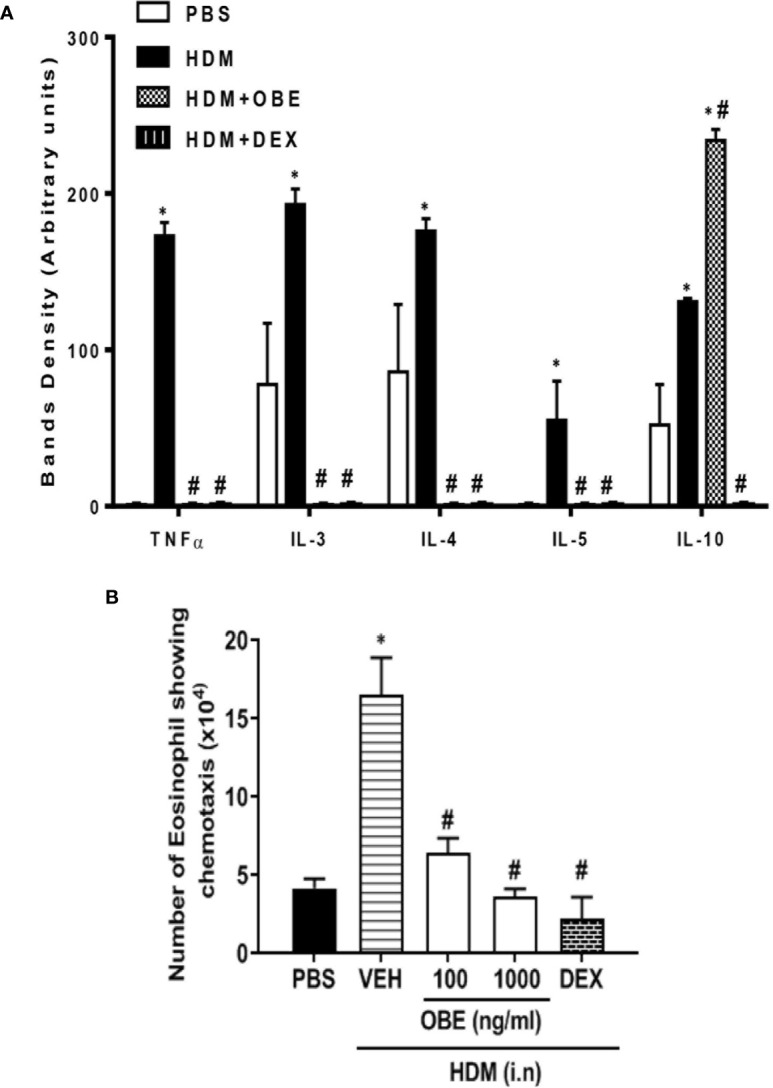
**(A)** Effect of OBE treatment (60 mg/kg; i.p.) on the airway expression of various pro-inflammatory cytokines. HDM challenge significantly enhanced the expression of the following interleukins (IL): IL-3, IL-4, IL-5, IL-10, and tumor necrosis factor (TNF-α). Treatment with OBE significantly reduced the expression of these cytokines except IL-10 which was significantly increased above the HDM levels. Data are expressed as mean ± SEM (n = 4). *P < 0.05 *vs* PBS group, ^#^P < 0.05 *vs* HDM group. **(B)** Effect of OBE (100 and 1,000 ng/ml) on HDM/BALF-induced eosinophil chemotaxis. BALF from HDM-challenged mice induced a significant increase in eosinophil chemotaxis compared to BALF from PBS-challenged mice. Pretreatment with OBE (100 and 1,000 ng/ml) dose-dependently inhibited eosinophil chemotaxis. Data are expressed as mean ± SEM (*n* = 5). *P < 0.05 *vs* PBS group, ^#^P < 0.05 *vs* HDM group.

The effect of OBE treatment (60 mg/kg) on the airway expression levels of various pro-inflammatory cytokines was determined. HDM challenge significantly enhanced the expression of the following interleukins (IL) by: IL-3 (147.4%), IL-4 (104.6%), IL-5 (5400%), IL-10 (152.0%), and tumor necrosis factor (TNF-α) (17,200%) (P < 0.05; **Figure 7A**, n = 4) compared to PBS-challenged mice. Treatment with OBE significantly reduced the expression of all of the above molecules by approximately 98.2–99.5%, except IL-10 (anti-inflammatory cytokine) which was significantly increased (78.6%) above the HDM levels (P < 0.05; **Figure 7A**, n = 4).

### Effect of OBE on Eosinophil Chemotaxis *Ex Vivo*

In this experiment, eosinophils showed significant migration towards BAL fluid derived from HDM-challenged mice compared to BAL fluid from PBS-challenged mice (16.5 ± 2.4 *vs* 4.2 ± 0.6 × 10^4^/ml, P < 0.05; **Figure 7B**). In contrast, pretreatment with OBE (100 and 1,000 ng/ml) dose-dependently inhibited the HDM/BAL fluid-induced eosinophil chemotaxis (6.4 ± 1.0 and 3.7 ± 0.5 *vs* 16.5 ± 2.4 × 10^4^/ml, respectively, P < 0.05; **Figure 7B**, n = 5).

### Effect of OBE on HDM-Induced Airway Hyperresponsiveness

**Figure 8:**
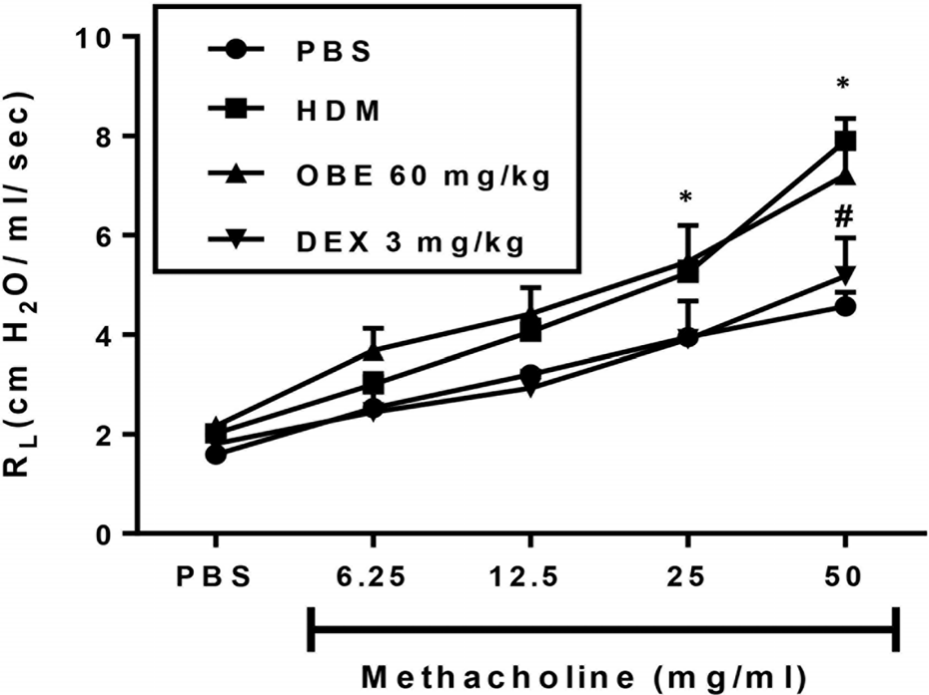
Effect of OBE treatment on HDM-induced AHR to inhaled methacholine. HDM challenged mice demonstrated significant AHR compared to the control group at doses 25 and 50 mg/kg of methacholine. Treatment with dexamethasone (3 mg/kg; i.p.) significantly reduced the HDM-induced AHR. However, treatment with OBE (60 mg/kg; i.p.) did not significantly reduce the average R_L_ in comparison with the HDM-challenged/vehicle-treated group at any of the doses of methacholine tested (P > 0.05). Data are expressed as mean ± SEM (*n* = 6–13). *P < 0.05 *vs* PBS group, ^#^P < 0.05 for HDM group *vs* DEX group.

In this experiment, we evaluated the effect of OBE treatment on the HDM-induced AHR (n = 6–13). Our data show that there was a significant increase in airway responsiveness 24 h after the last intranasal HDM challenge as demonstrated by a significant increase in lung resistance (R_L_) to methacholine in the HDM-challenged mice as compared to the PBS-treated control mice at a dose of 25 mg/ml (5.2 ± 0.2 *vs* 3.9 ± 0.2 cm H_2_O per ml/s) and 50 mg/ml (7.9 ± 0.5 *vs* 4.6 ± 0.3 cm H_2_O per ml/s) (**Figure 8**; P < 0.05). However, treatment with OBE did not significantly reduce the average R_L_ in comparison with the HDM-challenged/vehicle-treated group at any of the tested doses of methacholine (**Figure 8**; P > 0.05). Treatment with dexamethasone (3 mg/kg) nonetheless resulted in a significant reduction (7.9 ± 0.5 *vs* 5.2 ± 0.8 cm H_2_O per ml/s) (P < 0.05; **Figure 8**) of the HDM-induced AHR at the 50 mg/ml of methacholine.

### Synergism Between OBE and Dexamethasone on Airway Inflammatory Cell Influx

**Figure 9:**
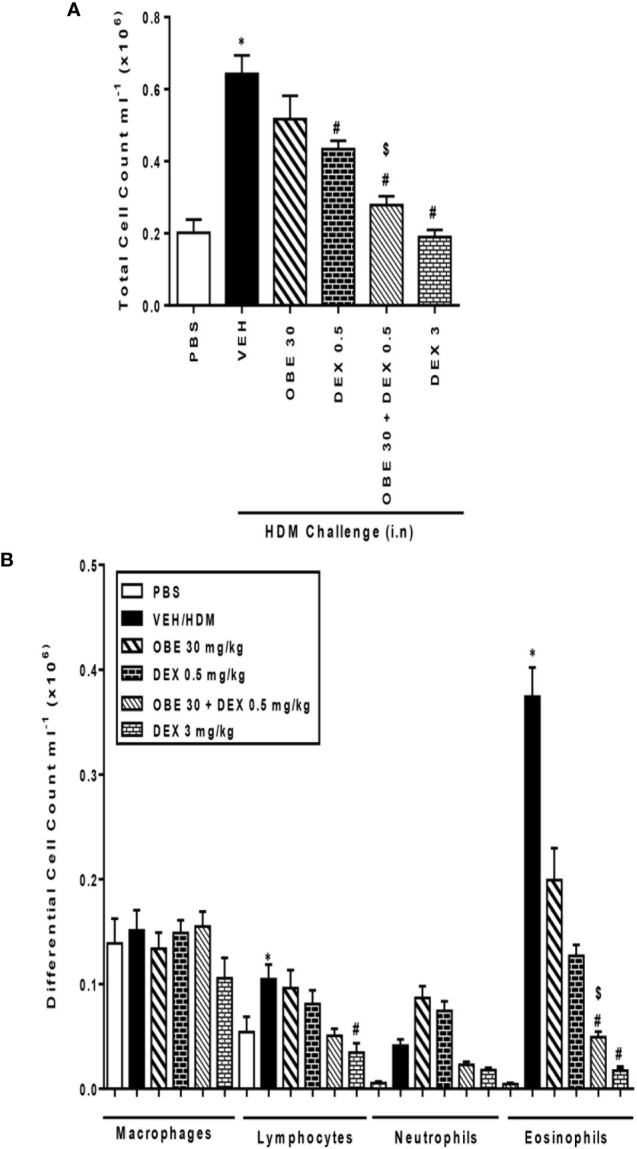
Effect of OBE (30 mg/kg; i.p.) both alone and in combination with low dexamethasone (0.5 mg/kg) on HDM-induced increase in **(A)** total cell and **(B)** differential cell count. Treatment with OBE, in combination with low dose dexamethasone, resulted in a significant inhibition of both total and eosinophil numbers compared to either treatment alone. Data are expressed as mean ± SEM (*n* = 7–11). *P < 0.05 *vs* PBS group, ^#^P < 0.05 *vs* HDM group and ^$^P* *< 0.05 *versus* either OBE (30 mg/kg; i.p.) alone or dexamethasone (0.5 mg/kg/i.p.) alone.

In this experiment, we evaluated the effect of combining OBE with dexamethasone on the HDM-induced total and differential cell influx. Treatment with either OBE 30 mg/kg or 0.5 mg/kg of dexamethasone had minimal to modest effects on the HDM-induced increase in the total cell count (5.2 ± 0.6 and 4.3 ± 0.2 *vs* 6.4 ± 0.5 × 10^5^, respectively, **Figure 9A**, n = 7–11). Furthermore, while treatment with either OBE at 30 mg/kg or dexamethasone at 0.5 mg/kg alone reduced the eosinophil influx, this did not reach statistical significance (OBE 30 and 0.5 mg/kg dexamethasone alone *vs* HDM, 1.9 ± 0.3 and 1.3 ± 0.1 *vs* 3.7 ± 0.3 × 10^5^, respectively, P > 0.05; **Figure 9B**). However, when OBE, at 30 mg/kg, was combined with dexamethasone [0.5 mg/kg], the inhibitory effect of this combination was significantly greater compared to either treatment when given alone (2.7 ± 0.2 *vs* 5.2 ± 0.6, 4.3 ± 0.2, 6.4 ± 0.5 × 10^5^, respectively, P < 0.05, **Figure 9A**) and was indeed comparable to the high dose dexamethasone (3 mg/kg). Similarly, the combined treatment of both OBE (30 mg/kg) and dexamethasone (0.5 mg/kg), resulted in a significant reduction in the HDM-induced airways eosinophilia (0.4 ± 0.1 *vs* 1.9 ± 0.3, 1.3 ± 0.1, 3.7 ± 0.3 × 10^5^, respectively, P < 0.05; **Figure 9B**) compared to either treament when given alone and was comparable to the high dose dexamethasone treatment (3 mg/kg).

### Synergism Between OBE and Dexamethasone on HDM-Induced Histopathological Changes

**Figure 10:**
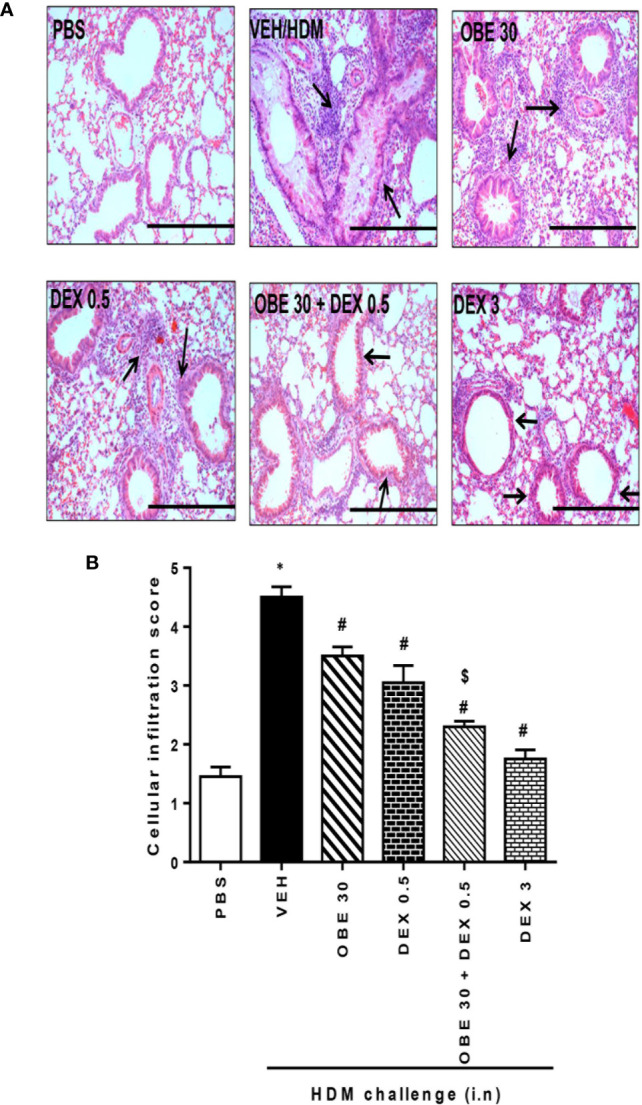
Effect of OBE (30 mg/kg; i.p.) alone and in combination with low dexamethasone (0.5 mg/kg) on HDM-induced peribronchial and perivascular inflammatory cell infiltrations. **(A)** Representative low-magnification light photomicrographs displaying H&E staining of whole lung samples from control PBS-challenged mice (PBS), HDM-challenged mice (HDM), HDM-challenged mice pretreated with OBE (30 mg/kg; i.p.) (OBE 30), HDM-challenged mice pretreated with low dexamethasone treated (0.5 mg/kg; i.p.) (DEX 0.5), HDM-challenged mice pretreated with a combination of OBE (30 mg/kg; i.p.) and low dexamethasone treated (0.5 mg/kg; i.p.) (DEX 30 + DEX 0.5), HDM-challenged mice pretreated with high dose dexamethasone (3 mg/kg; i.p.) (DEX 3), scale bar = 200 µm. Graphs shows **(B)** cellular infiltration score for H&E. OBE treatment in combination with low dose dexamethasone resulted in a significant decrease in both peribronchial and perivascular inflammatory compared to either treatment when give alone. Data are expressed as mean ± SEM (*n* = 5). *P < 0.05 *vs* PBS, ^#^P < 0.05 *vs* HDM and **^$^**P* *< 0.05 *versus* either OBE (30 mg/kg; i.p.) alone group or dexamethasone (0.5 mg/kg/i.p.) alone group.

**Figure 11:**
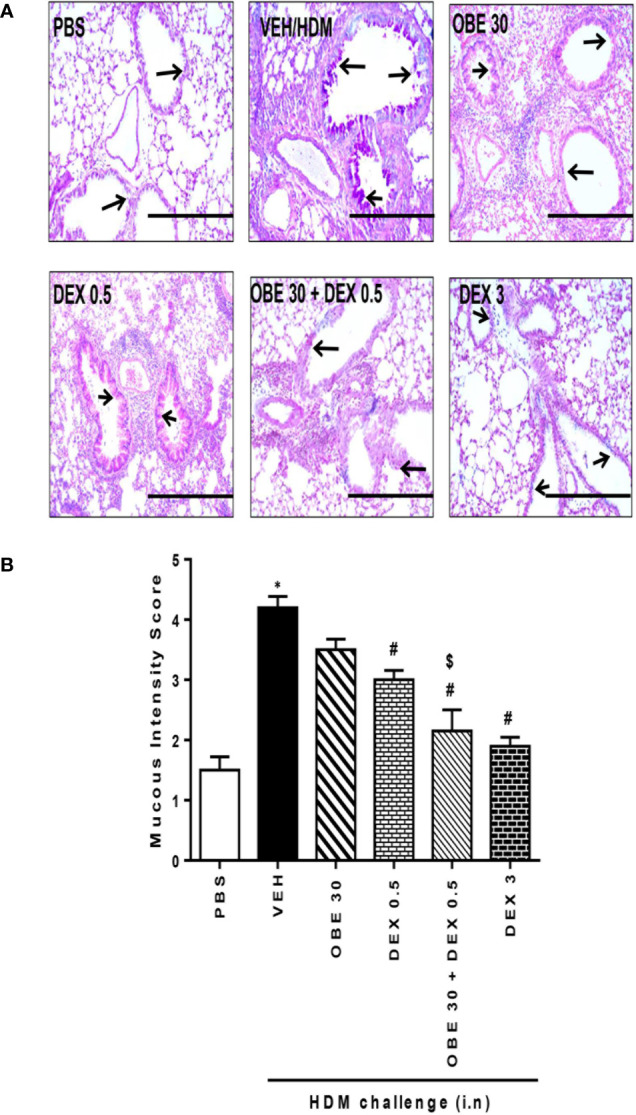
Effect of OBE (30 mg/kg; i.p.) both alone and in combination with low dexamethasone (0.5 mg/kg) on HDM-induced bronchial mucous production and goblet cell hyper/metaplasia. **(A)** Representative low-magnification light photomicrographs displaying PAS staining of whole lung samples from control PBS-challenged mice (PBS), HDM-challenged mice (HDM), HDM-challenged mice pretreated with OBE (30 mg/kg; i.p.) (OBE 30), HDM-challenged mice pretreated with low dose dexamethasone (0.5 mg/kg; i.p.) (DEX 0.5), HDM-challenged mice pretreated with a combination of OBE (30 mg/kg; i.p.) and low dose dexamethasone (0.5 mg/kg; i.p.) (OBE 30 + DEX 0.5), HDM-challenged mice pretreated with high dose dexamethasone (3 mg/kg; i.p.) (DEX 3), scale bar = 200µm. Graphs show **(B)** mucous intensity score. OBE treatment in combination with low dose dexamethasone resulted in a significant decrease in bronchial mucus production and goblet cell hyper/metaplasia compared to either treatment when give alone. Data are expressed as mean ± SEM (*n* = 5). *P < 0.05 *vs* PBS and ^#^P < 0.05 *vs* HDM and **^$^**P* *< 0.05 *versus* either OBE (30 mg/kg; i.p.) alone group or dexamethasone (0.5 mg/kg/i.p.) alone group.

In this experiment, our data show that treatment with either OBE (30 mg/kg) or dexamethasone (0.5 mg/kg) alone resulted in modest but significant reduction in the HDM-induced perivascular and peribronchial inflammation (cellular infiltration score; 3.5 ± 0.2 and 3.1 ± 0.3 *vs* 4.5 ± 0.2, respectively, P < 0.05; **Figures 10A, B**, n = 5). However, when OBE (30 mg/kg) was combined with dexamethasone (0.5 mg/kg), the inhibitory effect on the HDM-induced inflammation was now more marked and significantly greater than each treatment given alone (cellular infiltration score, 2.3 ± 0.1 *vs* 3.5 ± 0.2, 3.1 ± 0.3, 4.5 ± 0.2, respectively, P < 0.05; **Figures 10A, B**) and was similar to the high dose dexamethasone (3 mg/kg) treatment. Similarly, treatment with either OBE (30 mg/kg) or dexamethasone (0.5 mg/kg) alone resulted in a modest reduction in the HDM-induced goblet cell hyper/metaplasia and increase in bronchial mucus production (mucous intensity score; 3.5 ± 0.2 and 3.0 ± 0.2 *vs* 4.2 ± 0.2, respectively, P < 0.05; **Figures 11A, B**, n = 5). However, the combination treatment of OBE (30 mg/kg) with dexamethasone (0.5 mg) significantly inhibited the HDM-induced goblet cell hyper/metaplasia and increase in bronchial mucus production airway when compared to either OBE (30 mg/kg) or dexamethasone (0.5 mg/kg) given alone (mucous intensity score; 2.2 ± 0.4 *vs* 3.5 ± 0.2, 3.0 ± 0.2, 4.2 ± 0.2, respectively, P < 0.05; **Figures 11A, B**) and was almost as effective as dexamethasone at 3 mg/kg.

### Synergism Between OBE and Dexamethasone on HDM-Induced pERK1/2 Levels

**Figure 12:**
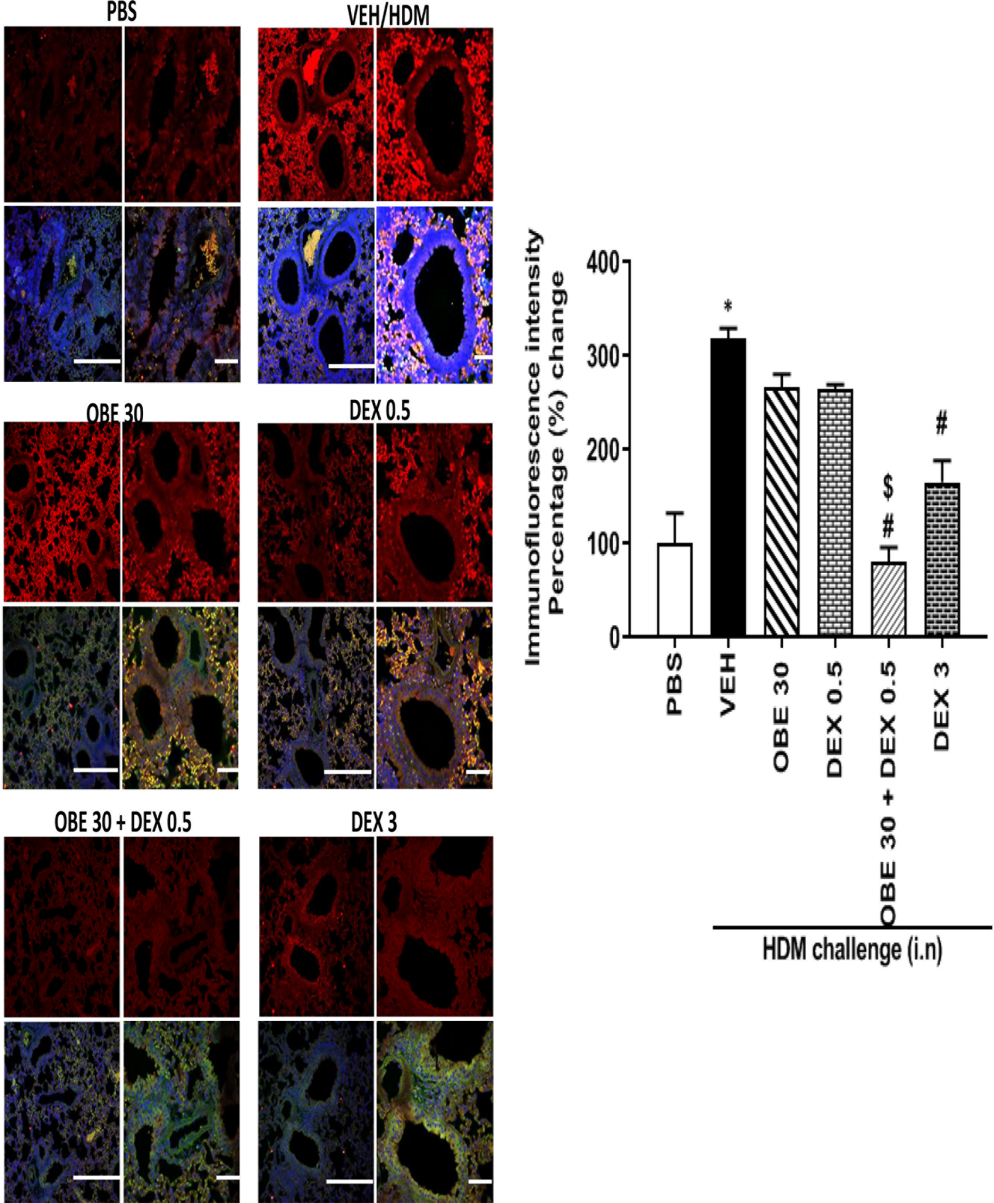
Immunofluorescence (Alexa Fluor) detection of phosphorylated ERK1/2 are shown in the upper panels overlaid with DAPI stain on the lower panel to show lung tissue architecture. Lung sections were taken from different treatment groups, control PBS-challenged mice (PBS), HDM-challenged mice (HDM), HDM-challenged mice pretreated with OBE (30 mg/kg; i.p.) (OBE 30), HDM-challenged mice pretreated with low dose dexamethasone (0.5 mg/kg; i.p.) (DEX 0.5), HDM-challenged mice pretreated with a combination of OBE (30 mg/kg; i.p.) and low dose dexamethasone (0.5 mg/kg; i.p.) (OBE 30 + DEX 0.5), HDM-challenged mice pretreated with high dose dexamethasone (3 mg/kg; i.p.) (DEX 3), scale bar = 50 µm. Graph shows quantitative assessment of fluorescence intensity of ERK (arbitrary units). Data are expressed as mean ± SEM (*n* = 4–5). *P < 0.05 *vs* PBS and ^#^P < 0.05 *vs* HDM and **^$^**P* *< 0.05 *versus* either OBE (30 mg/kg; i.p.) alone group or dexamethasone (0.5 mg/kg/i.p.) alone group.

In this experiment, our data show that treatment with either OBE (30 mg/kg) or the low dose dexamethasone (0.5 mg/kg) did not result in marked inhibition of the HDM-induced phosphorylation of ERK1/2 (267.0 ± 13.8 and 264.8 ± 4.9 *vs* 319.0 ± 10.4%, respectively, P < 0.05; **Figure 12**, n = 4–5). However, when OBE (30 mg/kg) was combined with the low dexamethasone (0.5 mg/kg), there was now a marked and significant reduction in pERK1/2 levels compared to either OBE (30 mg/kg) or the low dose dexamethasone (0.5 mg/kg) alone (80.0 ± 16.0 *vs* 267.0 ± 13.8 and 264.8 ± 4.9, respectively, P < 0.05; **Figure 12**). Of interest, the degree of inhibitory effect on pERK1/2, in the combination treatment, was twofold greater than that were obtained with the high dexamethasone dose (3 mg/kg) (P < 0.05; **Figure 12**).

## Discussion

The major finding of this study is that OBE produces anti-inflammatory actions in an established asthma model, partly, *via* inhibition of the EGFR/ERK1/2/AKT pathway. Furthermore, our data show that combined treatment with OBE and a classical steroid (dexamethasone) at sub-maximal doses resulted in an enhanced anti-inflammatory effect at the cellular, histopathological, and molecular levels.

The use of natural products as therapeutic agents has been on the rise, not only due to their demonstrated efficacy (both in humans and in preclinical models of disease) but possibly due to their perceived, and in many instances, real lack of adverse effects, even when consumed in large quantities (Bohlin et al., 2010; Atanasov et al., 2015). Based on this, many studies have been conducted over the past decades in an attempt to understand the scientific basis for the use of many natural products, elucidate their actions, and identify their mechanisms of action in animal models of disease (Fang et al., 2017). To this point, several studies have reported that OBE, or its constituents, have many pharmacological actions in many conditions and diseases such as wounds, scars, dysentery, inflammation, hypertension, and also asthma.

Our data show that OBE dose-dependently decreased the total and differential cell influx into the airways, at both 60 and 100 mg/kg dose, confirming data from recent studies reporting its inhibitory effect on BAL fluid cellularity (Ghorani et al., 2018). In line with this, we found that 60 mg/kg dose of OBE resulted in significant reduction of the HDM-induced perivascular and peribronchial inflammatory cell infiltration and goblet cell hyper/metaplasia. These anti-inflammatory actions were comparable to the action of dexamethasone (3 mg/kg) which indicates that the anti-inflammatory action of OBE is, at least, as effective as that of steroids. Our findings are in agreement with studies showing anti-inflammatory action of OBE in asthma-like models and more recently in a mouse model of colitis (Ghorani et al., 2018; Khajah et al., 2019). It is of interest to note that many of the documented activities for onion extract have been attributed to its polar constituents, particularly the known flavonoid quercetin, where polar solvents were mainly used to prepare the tested extracts (Oliveira et al., 2015). However, in this study, the anti-inflammatory action noted are most likely due to non-polar fat-soluble constituents, such as sulfur-containing compounds, mainly dipropyl disulfide and dipropyl trisulfide, which were found in abundance in the essential oil of onion since dichloromethane was used for the extraction method.

The EGF/EGFR is a critical signaling pathway in the pathogenesis of asthma, and both EGF and EGFR levels have been shown to be consistently increased in both human asthma and in animal models of asthma (Amishima et al., 1998; Puddicombe et al., 2000; Song L. et al., 2016). However, other ligands such as heparin-binding EGF-like growth factor (HB-EGF), amphiregulin, and betacellulin can also bind to and activate EGFR (Acciani et al., 2016; Ha and Rogers, 2016). In addition, ERK1/2 and AKT are not only key signaling molecules in asthma but have also been recently shown to be downstream of EGFR activation (El-Hashim et al., 2017). Our data show that treatment with OBE not only inhibited the development of asthma but also reduced pEGFR levels. Of interest and relevance is data from a recent study from our group, and that of others, using a similar model of asthma, has shown that treatment with selective EGFR inhibitors inhibited the EGFR-dependent signaling pathway and also reduced eosinophil recruitment, airway inflammation, AHR, and goblet cell hyper/metaplasia (Song L. et al., 2016; El-Hashim, 2017). Therefore, our findings would imply that the inhibitory effects of OBE on the asthmatic phenotype may be, at least partly, *via* inhibition of EGFR-dependent signaling perturbation. However, it is also likely that in addition to the EGFR pathway, OBE may inhibit other pro-inflammatory signaling pathways.

Our data also show that HDM-induced increase in pERK1/2 and pAKT were both inhibited following OBE treatment. While it is plausible that the effect of OBE on these signaling molecules represents independent and separate effects, since both ERK1/2 and AKT are ubiquitous signaling molecules, we have previously reported that AG1478, a selective EGFR receptor inhibitor, inhibited not only EGFR activation but also decreased pERK1/2 and pAKT levels, suggesting that both molecules are downstream of EGFR activation (El-Hashim et al., 2017). Therefore, it is very likely that the inhibitory effect of OBE on these two molecules is primarily due to upstream inhibition of EGFR-dependent signaling. This notion is further confirmed by our recent study showing that the effects of the anti-inflammatory endogenous molecule, Ang-(1-7), in asthma, are mediated *via* inhibition of the EGFR/ERK1/2 dependent signaling (El-Hashim et al., 2019). That OBE can inhibit the EGFR-triggered signaling perturbations suggests that it can inhibit a central signaling pathway in asthma.

It is well recognized that asthma is an immune response driven by Th2 and Th17 cells with cytokines such as IL-4, IL-5, IL-9, IL-10, IL-13, and IL-7 playing important roles (Saeki et al., 2019). Moreover, Th1 cytokines such as TNF-α and IFN-*γ* are also involved in asthma, with pro - and anti-asthma effects, respectively. Our findings show that OBE treatment resulted in a significant reduction of IL-4 and IL-5 consistent with other studies and effects of drugs that mediate an anti-allergic/anti-asthma action (Seo et al., 2019). In addition, OBE treatment decreased the pro-asthma TNF-α but increased the Th2 anti-inflammatory cytokine, IL-10. It is therefore plausible that the OBE-induced increase in IL-10 levels may, in part, explain the anti-inflammatory action of OBE in general and/or the specific decrease in the levels of IL-4 and IL-5, as these cytokines are known to have a reciprocal relationship (Oliveira et al., 2015).

The recent introduction of monoclonal antibodies targeting the eosinophil chemoattractant underscores the importance of eosinophils in asthma (Agache et al., 2020). Our data show that treatment with OBE significantly reduced eosinophil numbers not only in the tissue but also their *ex vivo* chemotaxis towards BAL fluid from HDM challenged mice. Studies have shown that onion constituents, such as thiosulfinates and cepaenes, dose-dependently inhibit both cyclooxygenase and 5-lipoxygenase enzyme activity (Werz, 2007). Furthermore, products of 5-lipoxygenase, namely the cysteinyl leukotrienes LTC_4_, LTD_4_, and LTE_4_, are known to be potent inducers of eosinophil chemotaxis (Werz, 2007). Therefore, inhibition of 5-lipoxygenase may represent one possible mechanism by which OBE inhibits eosinophil chemotaxis.

Our data also show that HDM challenge resulted in the induction of AHR, a characteristic feature of both clinical and preclinical asthma that is not easily amenable to asthma therapy (Busse, 2010; Ezeamuzie et al., 2014). Our findings show that treatment with OBE had no effect on this parameter. Although many studies have reported a causal link between airway inflammation and AHR, others have not been able to confirm this relationship, at least not for all types of AHR (Gozzard et al., 1996; Spina et al., 1998; El-Hashim et al., 1999; Spina and Page, 2009). Indeed, there may be a separation between the two phenomena, as not all agents that inhibit airway inflammation reduce AHR and *vice versa* (Riccio et al., 1997; El-Hashim et al., 2004). Recent studies have also provided good evidence that airway sensory hyper-excitability may also underlie AHR, and hyperactivity of airway nerves is less susceptible to anti-inflammatory agents (Spina and Page, 2009; Delescluse et al., 2012; Lommatzsch, 2012).

Although ICS have been the mainstay treatment for asthma, being effective anti-inflammatory agents, their major limitation is their high side effect profile, particularly with moderate-to-high doses (Hossny et al., 2016). In addition, the high cost of ICS is a real problem in poorer regions of the world (Watson and Lewis, 1997). To test whether OBE and steroids have a synergistic effect, we combined a low dose of OBE, 30 mg/kg and a low dose of dexamethasone, 0.5 mg/kg both of which produced minimal to mild anti-inflammatory effect when given alone. An important and novel finding in this study is that when OBE is combined with a low dose dexamethasone, a more powerful and effective anti-inflammatory effect was produced. Our data also show that the combination treatment resulted in a marked and significant enhancement of the anti-inflammatory effects on the total cell, eosinophil influx, histopathological changes (both perivascular and peribronchial inflammation and goblet cell hyper/metaplasia) when compared to either treatment alone. This combination was at least as effective as the high dose (3 mg/kg) steroid. To determine whether this was being mediated *via* enhanced suppression of the EGFR/ERK1/2/AKT signaling pathway, we assessed p-ERK1/2 expression level as a marker of activation of this signaling pathway. Our results clearly show that whilst neither 0.5 mg/kg dexamethasone nor 30 mg/kg alone had any effect on p-ERK1/2 levels, when both agents are combined, a significant and dramatic decrease in p-ERK1/2 levels was detected. This clearly indicates that the enhanced suppressive effects are being mediated *via* synergistic actions at the molecular level.

In conclusion, our data clearly show that OBE inhibits the asthma phenotype in a clinically relevant murine model of asthma, at least in part, *via* inhibition of Th2 cytokine profile, *via* inhibition of the EGFR/ERK/1/2/AKT signaling pathway. In addition, our data also show that combining OBE with steroids resulted in an enhanced anti-inflammatory effect *via* a synergistic action at the molecular signaling level. Therefore, this study not only identifies an important molecular signaling pathway that is targeted by OBE to inhibit the asthma phenotype, but also shows that OBE synergizes with steroids resulting in a greater anti-inflammatory action. This finding may have important implication for the treatment of asthma as it provides a potential to reduce steroid toxicity while maintaining efficacy.

## Data Availability Statement

The raw data supporting the conclusions of this article will be made available by the authors, without undue reservation.

## Ethics Statement

The animal study was reviewed and approved by Animal Welfare and Use of Laboratory Animals Committee in the Health Sciences Center.

## Author Contributions

Conceived and designed the experiments: AE-H, MK, and KO. Performed the experiments: SB, AA and HS. Analyzed the data: SB, AA, HS, AE-H, KO, and MK. Wrote the paper: AE-H. Edited the paper: KO and MK. All authors contributed to the article and approved the submitted version.

## Funding

This work was supported by Kuwait Foundation for the Advancement of Sciences, grant number: P11613PT01. Parts of this work were supported by grant SRUL02/13 to the Research Unit for Genomics, Proteomics and Cellomics Studies (OMICS), Kuwait University. GC-MS analyses were done at Research Sector Project Unit (RSPU), Faculty of Science, Kuwait University, supported by Grant numbers GS01/03.

## Conflict of Interest

The authors declare that the research was conducted in the absence of any commercial or financial relationships that could be construed as a potential conflict of interest.
